# The Old and the New

**Published:** 1874-12

**Authors:** 


*The Old and the New.*

We and you, kind readers, having traveled together in pleasant company for a whole twelve-month—climbing upon the one hand the rugged cliffs and mountain defiles of science; and, on the other, sauntering at will among the fresh blossoms and luscious fruits of medical literature, drinking from health-inspiring fountains of knowledge, tasting the ripe fruitage of experience, or idling cheerily along the devious walks of meditation and philosophy—having, we say, done all this to our heart's content, it is proper that we should all meet together for a moment in social chat, as the old year, burdened with its weight of ever-changeful days, sinks down upon the invisible border of a past eternity to rise no more forever, and the young year, beautiful and fresh as the first-born rose of June, leaps from the lap of mother Time to toy with the sceptre of the fallen king and proclaim himself the heir of the universe.

The seasons, in ceaseless round, have cheered us with their light, or oppressed us with their shadows, just as our hearts have felt the sunshine of pleasure or the gloom of pain and sorrow. We have had our beautiful visions and our holy dreams; we have felt the vanities of life; we have felt its beauty, its truth and its usefulness.

We have seen the smile upon our lips, we have felt the pang of disappointed hopes at our hearts. The violets have blossomed, and the snows are now falling upon the graves of dear ones, and the weight of their memory lies far heavier upon our spirits than the dark clods do that press their eyelids down in the dreamless chambers of death. We have seen friendship cast aside like a worthless bauble; we have seen the demons of hate, revenge and misanthropy released from their leashes to prey upon the unfortunate and the innocent; we have time and again realized the sad fact that there are spectres in every closet. But, dear and indulgent reader, does not the day follow the night? Do you not always find the fairest light upon the immediate verge of the darkest shadow? Is not life, our precious but woefully misunderstood human life, as changeful and capricious as an "April day?" Assuredly, and therefore we, if we have thankful hearts and justly balanced minds, must acknowledge the infinite blessings which the favor of our Heavenly Father has bestowed upon us during the past year, and the promises of blessings yet to come, perhaps to-morrow. We have had health, and hope, and pleasure, and schemes happily passed into successes; we hear the pleasant melody of our children's voices; the smile and the cheering voice of our life's companion is ours still; friendship and faith is not all a myth; truth conquers still; the beauty and harmony of nature still whisper to our heart of hearts the language of Heaven; peace drops her healing dews upon us, and the invisible hand of God's angels still guide us in love and patience to that shadowy bourne beyond which lie the green pastures and pleasant fields of our common immortality.

Then, most friendly reader, why should we mourn because the tomb builder, Time, has gathered the ashes of another mortal year into his vast receptacle?

The New Year stands ready like a brave young cavalier in the arena of the tournament to do battle for us, and break a lance in our cause. We cannot lift the veil of the future, it is true, but with the aid of fancy and a hopeful heart, we can paint the scenes that lie behind it in such a way that even our most dismal fears shall cease to haunt us, and every cloud assume its silver lining.

What say you, friends of the RECORD? "Here's a health to those that love us, and a smile for those who hate, and whatever skies above us," here's to each and all a merry Christmas and a happy New Year. P.

